# Clozapine and myopathic dysfunction: is creatinine kinase an important parameter to measure?

**DOI:** 10.1192/j.eurpsy.2022.2292

**Published:** 2022-09-01

**Authors:** P. Barbosa, M. Remelhe, Â. Venâncio, L. Ribeiro

**Affiliations:** 1 Centro Hospitalar de Vila Nova de Gaia/Espinho, Psychiatry, Vila Nova de Gaia, Portugal; 2 Centro Hospitalar de Vila Nova de Gaia, Psychiatry, Vila Nova de Gaia, Portugal; 3 Vila Nova de Gaia Hospital Center, Psychiatry And Mental Health Service, Vila Nova de Gaia, Portugal

**Keywords:** Myopathic Dysfunction, clozapine, CK elevation

## Abstract

**Introduction:**

There have been reports of myopathic dysfunction with creatinine kinase (CK) elevation associated with neuroleptics, particularly, in clozapine-treated patients. The patients in these reports did not have any other clinical symptoms or signs indicative of neuropleptic malignant syndrome. Myopathic dysfunction was supported by the presence of CK elevations and either proximal limb weakness or fatigue, characteristic electrophysiologic abnormalities, or both. It has also been reported that CK elevation is neither dose nor treatment-duration dependent. The underlying mechanism is still unknown although it has been postulated that it is associated with cytochrome P450 interactions and/or calmodulin antagonism.

**Objectives:**

To report a case of elevation of CK during treatment with clozapine.

**Methods:**

The authors report a case of elevation of CK during treatment with clozapine. A non-systematic review was conducted by searching the PubMed database, using the terms “clozapine”, “myotoxicity”.

**Results:**

A 36-year-old man was admitted after abandoning treatment with clozapine. During the titration of the medication, he developed complaints of muscle fatigue in conjunction with an elevation of CK. CK levels normalized after intravenous hydration and with dose reduction. Furthermore, CK levels would increase with clozapine re-titration.

**Conclusions:**

Clozapine has numerous side effects. Myopathic dysfunction with CK elevation is a possible side effect which could have serious consequences such as renal impairment. In such cases, appropriate treatment should be implemented. Therefore, clinicians should be aware of this potential side effect.

**Disclosure:**

No significant relationships.

